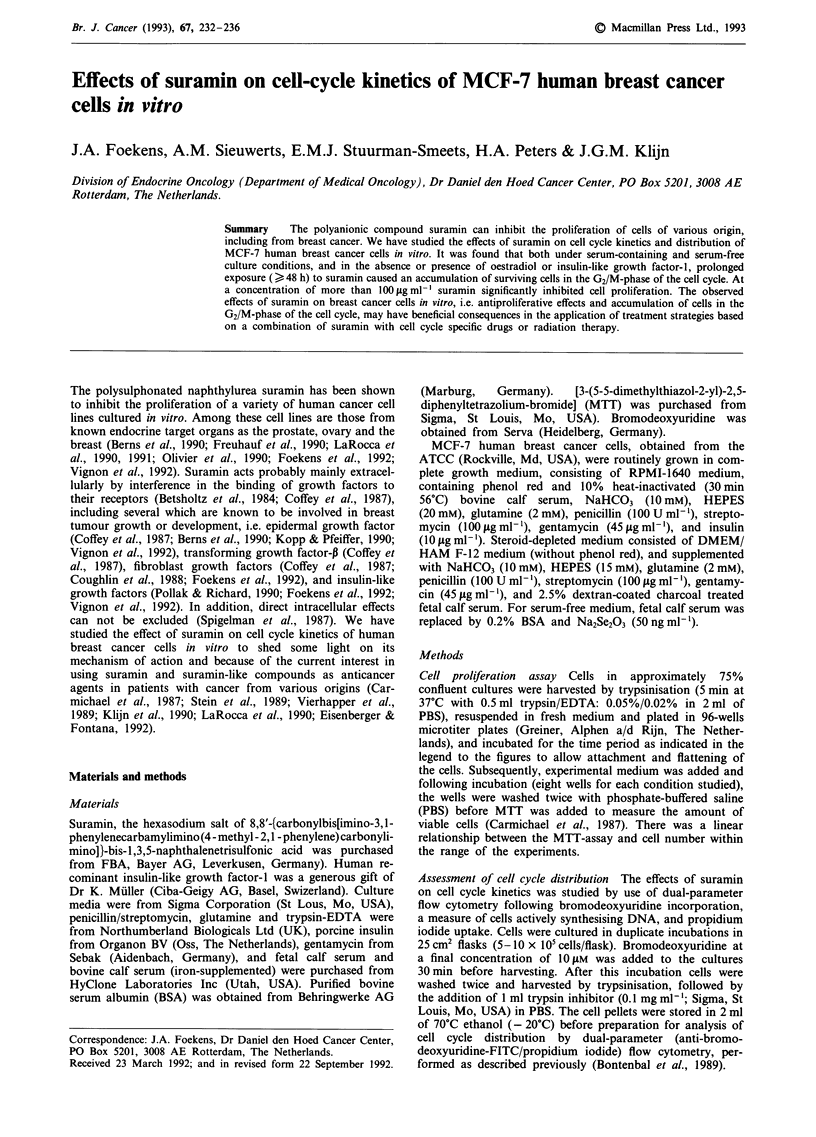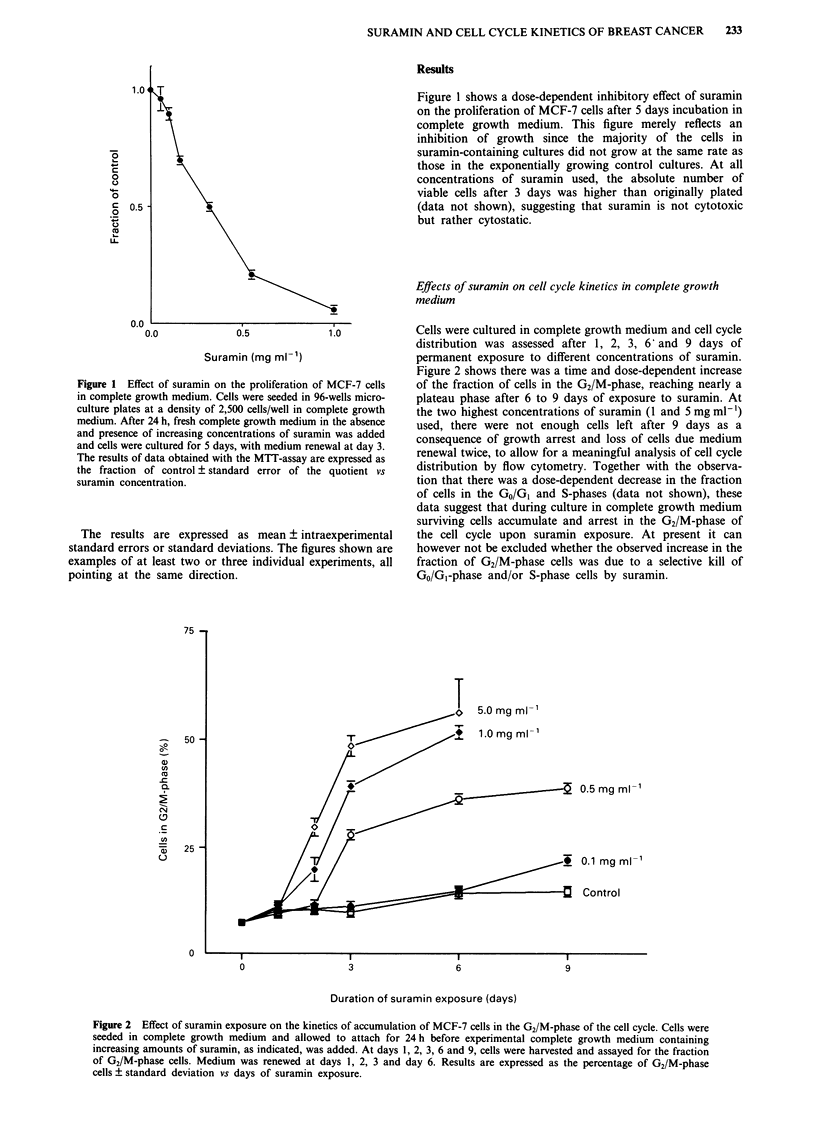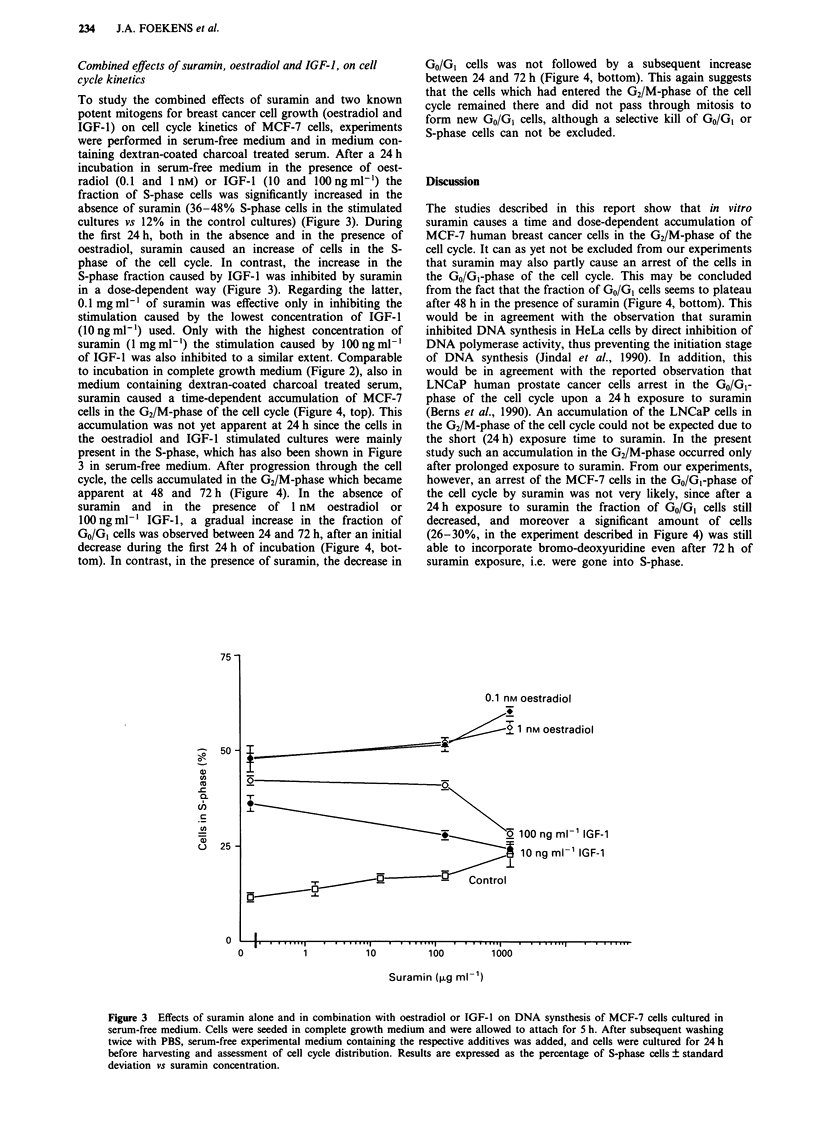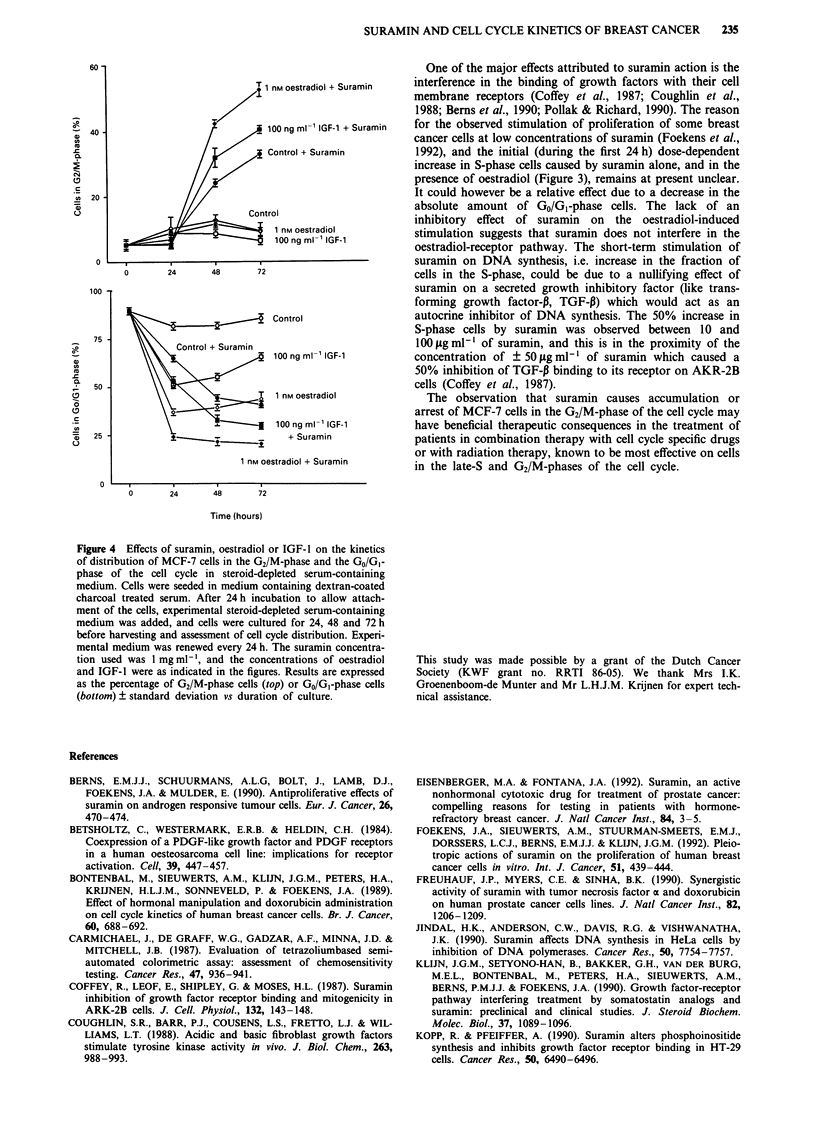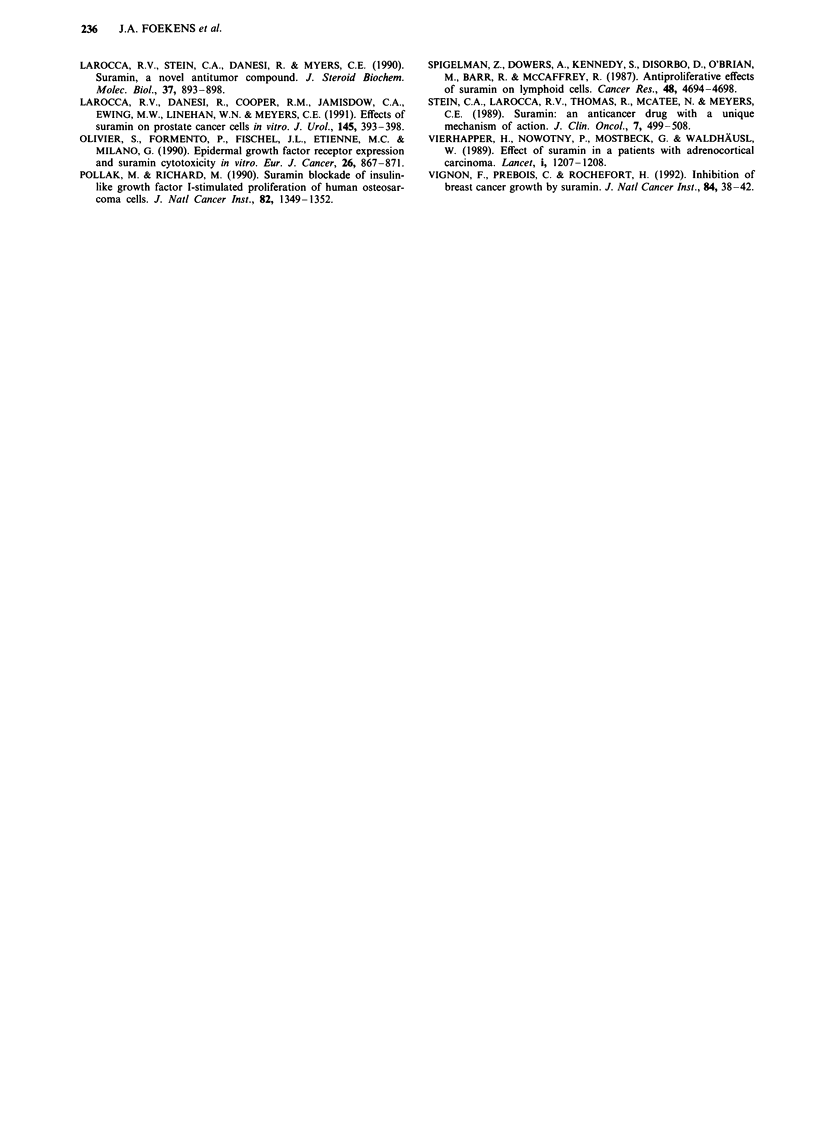# Effects of suramin on cell-cycle kinetics of MCF-7 human breast cancer cells in vitro.

**DOI:** 10.1038/bjc.1993.45

**Published:** 1993-02

**Authors:** J. A. Foekens, A. M. Sieuwerts, E. M. Stuurman-Smeets, H. A. Peters, J. G. Klijn

**Affiliations:** Department of Medical Oncology, Dr Daniel den Hoed Cancer Center, Rotterdam, The Netherlands.

## Abstract

The polyanionic compound suramin can inhibit the proliferation of cells of various origin, including from breast cancer. We have studied the effects of suramin on cell cycle kinetics and distribution of MCF-7 human breast cancer cells in vitro. It was found that both under serum-containing and serum-free culture conditions, and in the absence or presence of oestradiol or insulin-like growth factor-1, prolonged exposure (> or = 48 h) to suramin caused an accumulation of surviving cells in the G2/M-phase of the cell cycle. At a concentration of more than 100 micrograms ml-1 suramin significantly inhibited cell proliferation. The observed effects of suramin on breast cancer cells in vitro, i.e. antiproliferative effects and accumulation of cells in the G2/M-phase of the cell cycle, may have beneficial consequences in the application of treatment strategies based on a combination of suramin with cell cycle specific drugs or radiation therapy.


Br. J. Cancer (1993), 67, 232-236 © Macmillan Press Ltd., 1993

### Effects of suramin on cell-cycle kinetics of MCF-7 human breast cancer cells *in vitro*

J.A. Foekens, A.M. Sieuwerts, E.M.J. Stuurman-Smeets, H.A. Peters & J.G.M. Klijn

*Division of Endocrine Oncology (Department of Medical Oncology), Dr Daniel den Hoed Cancer Center, PO Box 5201, 3008 AE Rotterdam, The Netherlands.*

**Summary** The polyanionic compound suramin can inhibit the proliferation of cells of various origin, including from breast cancer. We have studied the effects of suramin on cell cycle kinetics and distribution of MCF-7 human breast cancer cells *in vitro*. It was found that both under serum-containing and serum-free culture conditions, and in the absence or presence of oestradiol or insulin-like growth factor-1, prolonged exposure ($\geqslant$48 h) to suramin caused an accumulation of surviving cells in the $G_2$/M-phase of the cell cycle. At a concentration of more than 100 $\mu g\,ml^{-1}$ suramin significantly inhibited cell proliferation. The observed effects of suramin on breast cancer cells *in vitro*, i.e. antiproliferative effects and accumulation of cells in the $G_2$/M-phase of the cell cycle, may have beneficial consequences in the application of treatment strategies based on a combination of suramin with cell cycle specific drugs or radiation therapy.

The polysulphonated naphthylurea suramin has been shown to inhibit the proliferation of a variety of human cancer cell lines cultured *in vitro*. Among these cell lines are those from known endocrine target organs as the prostate, ovary and the breast (Berns *et al.*, 1990; Freuhauf *et al.*, 1990; LaRocca *et al.*, 1990, 1991; Olivier *et al.*, 1990; Foekens *et al.*, 1992; Vignon *et al.*, 1992). Suramin acts probably mainly extracellularly by interference in the binding of growth factors to their receptors (Betsholtz *et al.*, 1984; Coffey *et al.*, 1987), including several which are known to be involved in breast tumour growth or development, i.e. epidermal growth factor (Coffey *et al.*, 1987; Berns *et al.*, 1990; Kopp & Pfeiffer, 1990; Vignon *et al.*, 1992), transforming growth factor-$\beta$ (Coffey *et al.*, 1987), fibroblast growth factors (Coffey *et al.*, 1987; Coughlin *et al.*, 1988; Foekens *et al.*, 1992), and insulin-like growth factors (Pollak & Richard, 1990; Foekens *et al.*, 1992; Vignon *et al.*, 1992). In addition, direct intracellular effects can not be excluded (Spigelman *et al.*, 1987). We have studied the effect of suramin on cell cycle kinetics of human breast cancer cells *in vitro* to shed some light on its mechanism of action and because of the current interest in using suramin and suramin-like compounds as anticancer agents in patients with cancer from various origins (Carmichael *et al.*, 1987; Stein *et al.*, 1989; Vierhapper *et al.*, 1989; Klijn *et al.*, 1990; LaRocca *et al.*, 1990; Eisenberger & Fontana, 1992).

### Materials and methods

### Materials

Suramin, the hexasodium salt of 8,8′-{carbonylbis[imino-3,1-phenylenecarbamylimino(4-methyl-2,1-phenylene)carbonylimino]}-bis-1,3,5-naphthalenetrisulfonic acid was purchased from FBA, Bayer AG, Leverkusen, Germany). Human recominant insulin-like growth factor-1 was a generous gift of Dr K. Müller (Ciba-Geigy AG, Basel, Swizerland). Culture media were from Sigma Corporation (St Lous, Mo, USA), penicillin/streptomycin, glutamine and trypsin-EDTA were from Northumberland Biologicals Ltd (UK), porcine insulin from Organon BV (Oss, The Netherlands), gentamycin from Sebak (Aidenbach, Germany), and fetal calf serum and bovine calf serum (iron-supplemented) were purchased from HyClone Laboratories Inc (Utah, USA). Purified bovine serum albumin (BSA) was obtained from Behringwerke AG (Marburg, Germany). [3-(5-5-dimethylthiazol-2-yl)-2,5-diphenyltetrazolium-bromide] (MTT) was purchased from Sigma, St Louis, Mo, USA). Bromodeoxyuridine was obtained from Serva (Heidelberg, Germany).

MCF-7 human breast cancer cells, obtained from the ATCC (Rockville, Md, USA), were routinely grown in complete growth medium, consisting of RPMI-1640 medium, containing phenol red and 10% heat-inactivated (30 min 56°C) bovine calf serum, $NaHCO_3$ (10 mM), HEPES (20 mM), glutamine (2 mM), penicillin (100 U $ml^{-1}$), streptomycin (100 $\mu g\,ml^{-1}$), gentamycin (45 $\mu g\,ml^{-1}$), and insulin (10 $\mu g\,ml^{-1}$). Steroid-depleted medium consisted of DMEM/HAM F-12 medium (without phenol red), and supplemented with $NaHCO_3$ (10 mM), HEPES (15 mM), glutamine (2 mM), penicillin (100 U $ml^{-1}$), streptomycin (100 $\mu g\,ml^{-1}$), gentamycin (45 $\mu g\,ml^{-1}$), and 2.5% dextran-coated charcoal treated fetal calf serum. For serum-free medium, fetal calf serum was replaced by 0.2% BSA and $Na_2Se_2O_3$ (50 ng $ml^{-1}$).

### Methods

*Cell proliferation assay* Cells in approximately 75% confluent cultures were harvested by trypsinisation (5 min at 37°C with 0.5 ml trypsin/EDTA: 0.05%/0.02% in 2 ml of PBS), resuspended in fresh medium and plated in 96-wells microtiter plates (Greiner, Alphen a/d Rijn, The Netherlands), and incubated for the time period as indicated in the legend to the figures to allow attachment and flattening of the cells. Subsequently, experimental medium was added and following incubation (eight wells for each condition studied), the wells were washed twice with phosphate-buffered saline (PBS) before MTT was added to measure the amount of viable cells (Carmichael *et al.*, 1987). There was a linear relationship between the MTT-assay and cell number within the range of the experiments.

*Assessment of cell cycle distribution* The effects of suramin on cell cycle kinetics was studied by use of dual-parameter flow cytometry following bromodeoxyuridine incorporation, a measure of cells actively synthesising DNA, and propidium iodide uptake. Cells were cultured in duplicate incubations in 25 $cm^2$ flasks (5–10 × $10^5$ cells/flask). Bromodeoxyuridine at a final concentration of 10 $\mu$M was added to the cultures 30 min before harvesting. After this incubation cells were washed twice and harvested by trypsinisation, followed by the addition of 1 ml trypsin inhibitor (0.1 mg $ml^{-1}$; Sigma, St Louis, Mo, USA) in PBS. The cell pellets were stored in 2 ml of 70°C ethanol (− 20°C) before preparation for analysis of cell cycle distribution by dual-parameter (anti-bromodeoxyuridine-FITC/propidium iodide) flow cytometry, performed as described previously (Bontenbal *et al.*, 1989).

Correspondence: J.A. Foekens, Dr Daniel den Hoed Cancer Center, PO Box 5201, 3008 AE Rotterdam, The Netherlands.
Received 23 March 1992; and in revised form 22 September 1992.

**Figure 1** Effect of suramin on the proliferation of MCF-7 cells in complete growth medium. Cells were seeded in 96-wells microculture plates at a density of 2,500 cells/well in complete growth medium. After 24 h, fresh complete growth medium in the absence and presence of increasing concentrations of suramin was added and cells were cultured for 5 days, with medium renewal at day 3. The results of data obtained with the MTT-assay are expressed as the fraction of control ± standard error of the quotient *vs* suramin concentration.

The results are expressed as mean ± intraexperimental standard errors or standard deviations. The figures shown are examples of at least two or three individual experiments, all pointing at the same direction.

### Results

Figure 1 shows a dose-dependent inhibitory effect of suramin on the proliferation of MCF-7 cells after 5 days incubation in complete growth medium. This figure merely reflects an inhibition of growth since the majority of the cells in suramin-containing cultures did not grow at the same rate as those in the exponentially growing control cultures. At all concentrations of suramin used, the absolute number of viable cells after 3 days was higher than originally plated (data not shown), suggesting that suramin is not cytotoxic but rather cytostatic.

### *Effects of suramin on cell cycle kinetics in complete growth medium*

Cells were cultured in complete growth medium and cell cycle distribution was assessed after 1, 2, 3, 6' and 9 days of permanent exposure to different concentrations of suramin. Figure 2 shows there was a time and dose-dependent increase of the fraction of cells in the $G_2$/M-phase, reaching nearly a plateau phase after 6 to 9 days of exposure to suramin. At the two highest concentrations of suramin (1 and 5 mg ml$^{-1}$) used, there were not enough cells left after 9 days as a consequence of growth arrest and loss of cells due medium renewal twice, to allow for a meaningful analysis of cell cycle distribution by flow cytometry. Together with the observation that there was a dose-dependent decrease in the fraction of cells in the $G_0$/$G_1$ and S-phases (data not shown), these data suggest that during culture in complete growth medium surviving cells accumulate and arrest in the $G_2$/M-phase of the cell cycle upon suramin exposure. At present it can however not be excluded whether the observed increase in the fraction of $G_2$/M-phase cells was due to a selective kill of $G_0$/$G_1$-phase and/or S-phase cells by suramin.

**Figure 2** Effect of suramin exposure on the kinetics of accumulation of MCF-7 cells in the $G_2$/M-phase of the cell cycle. Cells were seeded in complete growth medium and allowed to attach for 24 h before experimental complete growth medium containing increasing amounts of suramin, as indicated, was added. At days 1, 2, 3, 6 and 9, cells were harvested and assayed for the fraction of $G_2$/M-phase cells. Medium was renewed at days 1, 2, 3 and day 6. Results are expressed as the percentage of $G_2$/M-phase cells ± standard deviation *vs* days of suramin exposure.

*Combined effects of suramin, oestradiol and IGF-1, on cell cycle kinetics*

To study the combined effects of suramin and two known potent mitogens for breast cancer cell growth (oestradiol and IGF-1) on cell cycle kinetics of MCF-7 cells, experiments were performed in serum-free medium and in medium containing dextran-coated charcoal treated serum. After a 24 h incubation in serum-free medium in the presence of oestradiol (0.1 and 1 nM) or IGF-1 (10 and 100 ng $ml^{-1}$) the fraction of S-phase cells was significantly increased in the absence of suramin (36–48% S-phase cells in the stimulated cultures *vs* 12% in the control cultures) (Figure 3). During the first 24 h, both in the absence and in the presence of oestradiol, suramin caused an increase of cells in the S-phase of the cell cycle. In contrast, the increase in the S-phase fraction caused by IGF-1 was inhibited by suramin in a dose-dependent way (Figure 3). Regarding the latter, 0.1 mg $ml^{-1}$ of suramin was effective only in inhibiting the stimulation caused by the lowest concentration of IGF-1 (10 ng $ml^{-1}$) used. Only with the highest concentration of suramin (1 mg $ml^{-1}$) the stimulation caused by 100 ng $ml^{-1}$ of IGF-1 was also inhibited to a similar extent. Comparable to incubation in complete growth medium (Figure 2), also in medium containing dextran-coated charcoal treated serum, suramin caused a time-dependent accumulation of MCF-7 cells in the $G_2$/M-phase of the cell cycle (Figure 4, top). This accumulation was not yet apparent at 24 h since the cells in the oestradiol and IGF-1 stimulated cultures were mainly present in the S-phase, which has also been shown in Figure 3 in serum-free medium. After progression through the cell cycle, the cells accumulated in the $G_2$/M-phase which became apparent at 48 and 72 h (Figure 4). In the absence of suramin and in the presence of 1 nM oestradiol or 100 ng $ml^{-1}$ IGF-1, a gradual increase in the fraction of $G_0/G_1$ cells was observed between 24 and 72 h, after an initial decrease during the first 24 h of incubation (Figure 4, bottom). In contrast, in the presence of suramin, the decrease in $G_0/G_1$ cells was not followed by a subsequent increase between 24 and 72 h (Figure 4, bottom). This again suggests that the cells which had entered the $G_2$/M-phase of the cell cycle remained there and did not pass through mitosis to form new $G_0/G_1$ cells, although a selective kill of $G_0/G_1$ or S-phase cells can not be excluded.

### Discussion

The studies described in this report show that *in vitro* suramin causes a time and dose-dependent accumulation of MCF-7 human breast cancer cells in the $G_2$/M-phase of the cell cycle. It can as yet not be excluded from our experiments that suramin may also partly cause an arrest of the cells in the $G_0/G_1$-phase of the cell cycle. This may be concluded from the fact that the fraction of $G_0/G_1$ cells seems to plateau after 48 h in the presence of suramin (Figure 4, bottom). This would be in agreement with the observation that suramin inhibited DNA synthesis in HeLa cells by direct inhibition of DNA polymerase activity, thus preventing the initiation stage of DNA synthesis (Jindal *et al.*, 1990). In addition, this would be in agreement with the reported observation that LNCaP human prostate cancer cells arrest in the $G_0/G_1$-phase of the cell cycle upon a 24 h exposure to suramin (Berns *et al.*, 1990). An accumulation of the LNCaP cells in the $G_2$/M-phase of the cell cycle could not be expected due to the short (24 h) exposure time to suramin. In the present study such an accumulation in the $G_2$/M-phase occurred only after prolonged exposure to suramin. From our experiments, however, an arrest of the MCF-7 cells in the $G_0/G_1$-phase of the cell cycle by suramin was not very likely, since after a 24 h exposure to suramin the fraction of $G_0/G_1$ cells still decreased, and moreover a significant amount of cells (26–30%, in the experiment described in Figure 4) was still able to incorporate bromo-deoxyuridine even after 72 h of suramin exposure, i.e. were gone into S-phase.

**Figure 3** Effects of suramin alone and in combination with oestradiol or IGF-1 on DNA synsthesis of MCF-7 cells cultured in serum-free medium. Cells were seeded in complete growth medium and were allowed to attach for 5 h. After subsequent washing twice with PBS, serum-free experimental medium containing the respective additives was added, and cells were cultured for 24 h before harvesting and assessment of cell cycle distribution. Results are expressed as the percentage of S-phase cells ± standard deviation *vs* suramin concentration.

**Figure 4** Effects of suramin, oestradiol or IGF-1 on the kinetics of distribution of MCF-7 cells in the $G_2$/M-phase and the $G_0/G_1$-phase of the cell cycle in steroid-depleted serum-containing medium. Cells were seeded in medium containing dextran-coated charcoal treated serum. After 24 h incubation to allow attachment of the cells, experimental steroid-depleted serum-containing medium was added, and cells were cultured for 24, 48 and 72 h before harvesting and assessment of cell cycle distribution. Experimental medium was renewed every 24 h. The suramin concentration used was 1 mg ml$^{-1}$, and the concentrations of oestradiol and IGF-1 were as indicated in the figures. Results are expressed as the percentage of $G_2$/M-phase cells (*top*) or $G_0/G_1$-phase cells (*bottom*) ± standard deviation *vs* duration of culture.

One of the major effects attributed to suramin action is the interference in the binding of growth factors with their cell membrane receptors (Coffey *et al.*, 1987; Coughlin *et al.*, 1988; Berns *et al.*, 1990; Pollak & Richard, 1990). The reason for the observed stimulation of proliferation of some breast cancer cells at low concentrations of suramin (Foekens *et al.*, 1992), and the initial (during the first 24 h) dose-dependent increase in S-phase cells caused by suramin alone, and in the presence of oestradiol (Figure 3), remains at present unclear. It could however be a relative effect due to a decrease in the absolute amount of $G_0/G_1$-phase cells. The lack of an inhibitory effect of suramin on the oestradiol-induced stimulation suggests that suramin does not interfere in the oestradiol-receptor pathway. The short-term stimulation of suramin on DNA synthesis, i.e. increase in the fraction of cells in the S-phase, could be due to a nullifying effect of suramin on a secreted growth inhibitory factor (like transforming growth factor-$\beta$, TGF-$\beta$) which would act as an autocrine inhibitor of DNA synthesis. The 50% increase in S-phase cells by suramin was observed between 10 and 100 $\mu$g ml$^{-1}$ of suramin, and this is in the proximity of the concentration of ± 50 $\mu$g ml$^{-1}$ of suramin which caused a 50% inhibition of TGF-$\beta$ binding to its receptor on AKR-2B cells (Coffey *et al.*, 1987).

The observation that suramin causes accumulation or arrest of MCF-7 cells in the $G_2$/M-phase of the cell cycle may have beneficial therapeutic consequences in the treatment of patients in combination therapy with cell cycle specific drugs or with radiation therapy, known to be most effective on cells in the late-S and $G_2$/M-phases of the cell cycle.

This study was made possible by a grant of the Dutch Cancer Society (KWF grant no. RRTI 86-05). We thank Mrs I.K. Groenenboom-de Munter and Mr L.H.J.M. Krijnen for expert technical assistance.

### References

BERNS, E.M.J.J., SCHUURMANS, A.L.G, BOLT, J., LAMB, D.J., FOEKENS, J.A. & MULDER, E. (1990). Antiproliferative effects of suramin on androgen responsive tumour cells. *Eur. J. Cancer*, **26**, 470-474.

BETSHOLTZ, C., WESTERMARK, E.R.B. & HELDIN, C.H. (1984). Coexpression of a PDGF-like growth factor and PDGF receptors in a human oesteosarcoma cell line: implications for receptor activation. *Cell*, **39**, 447-457.

BONTENBAL, M., SIEUWERTS, A.M., KLIJN, J.G.M., PETERS, H.A., KRIJNEN, H.L.J.M., SONNEVELD, P. & FOEKENS, J.A. (1989). Effect of hormonal manipulation and doxorubicin administration on cell cycle kinetics of human breast cancer cells. *Br. J. Cancer*, **60**, 688-692.

CARMICHAEL, J., DE GRAFF, W.G., GADZAR, A.F., MINNA, J.D. & MITCHELL, J.B. (1987). Evaluation of tetrazoliumbased semi-automated colorimetric assay: assessment of chemosensitivity testing. *Cancer Res.*, **47**, 936-941.

COFFEY, R., LEOF, E., SHIPLEY, G. & MOSES, H.L. (1987). Suramin inhibition of growth factor receptor binding and mitogenicity in ARK-2B cells. *J. Cell. Physiol.*, **132**, 143-148.

COUGHLIN, S.R., BARR, P.J., COUSENS, L.S., FRETTO, L.J. & WILLIAMS, L.T. (1988). Acidic and basic fibroblast growth factors stimulate tyrosine kinase activity *in vivo*. *J. Biol. Chem.*, **263**, 988-993.

EISENBERGER, M.A. & FONTANA, J.A. (1992). Suramin, an active nonhormonal cytotoxic drug for treatment of prostate cancer: compelling reasons for testing in patients with hormone-refractory breast cancer. *J. Natl Cancer Inst.*, **84**, 3-5.

FOEKENS, J.A., SIEUWERTS, A.M., STUURMAN-SMEETS, E.M.J., DORSSERS, L.C.J., BERNS, E.M.J.J. & KLIJN, J.G.M. (1992). Pleiotropic actions of suramin on the proliferation of human breast cancer cells *in vitro*. *Int. J. Cancer*, **51**, 439-444.

FREUHAUF, J.P., MYERS, C.E. & SINHA, B.K. (1990). Synergistic activity of suramin with tumor necrosis factor α and doxorubicin on human prostate cancer cells lines. *J. Natl Cancer Inst.*, **82**, 1206-1209.

JINDAL, H.K., ANDERSON, C.W., DAVIS, R.G. & VISHWANATHA, J.K. (1990). Suramin affects DNA synthesis in HeLa cells by inhibition of DNA polymerases. *Cancer Res.*, **50**, 7754-7757.

KLIJN, J.G.M., SETYONO-HAN, B., BAKKER, G.H., VAN DER BURG, M.E.L., BONTENBAL, M., PETERS, H.A., SIEUWERTS, A.M., BERNS, P.M.J.J. & FOEKENS, J.A. (1990). Growth factor-receptor pathway interfering treatment by somatostatin analogs and suramin: preclinical and clinical studies. *J. Steroid Biochem. Molec. Biol.*, **37**, 1089-1096.

KOPP, R. & PFEIFFER, A. (1990). Suramin alters phosphoinositide synthesis and inhibits growth factor receptor binding in HT-29 cells. *Cancer Res.*, **50**, 6490-6496.

LAROCCA, R.V., STEIN, C.A., DANESI, R. & MYERS, C.E. (1990). Suramin, a novel antitumor compound. *J. Steroid Biochem. Molec. Biol.*, **37**, 893–898.

LAROCCA, R.V., DANESI, R., COOPER, R.M., JAMISDOW, C.A., EWING, M.W., LINEHAN, W.N. & MEYERS, C.E. (1991). Effects of suramin on prostate cancer cells *in vitro*. *J. Urol.*, **145**, 393–398.

OLIVIER, S., FORMENTO, P., FISCHEL, J.L., ETIENNE, M.C. & MILANO, G. (1990). Epidermal growth factor receptor expression and suramin cytotoxicity *in vitro*. *Eur. J. Cancer*, **26**, 867–871.

POLLAK, M. & RICHARD, M. (1990). Suramin blockade of insulin-like growth factor I-stimulated proliferation of human osteosarcoma cells. *J. Natl Cancer Inst.*, **82**, 1349–1352.

SPIGELMAN, Z., DOWERS, A., KENNEDY, S., DISORBO, D., O'BRIAN, M., BARR, R. & MCCAFFREY, R. (1987). Antiproliferative effects of suramin on lymphoid cells. *Cancer Res.*, **48**, 4694–4698.

STEIN, C.A., LAROCCA, R.V., THOMAS, R., MCATEE, N. & MEYERS, C.E. (1989). Suramin: an anticancer drug with a unique mechanism of action. *J. Clin. Oncol.*, **7**, 499–508.

VIERHAPPER, H., NOWOTNY, P., MOSTBECK, G. & WALDHÄUSL, W. (1989). Effect of suramin in a patients with adrenocortical carcinoma. *Lancet*, **i**, 1207–1208.

VIGNON, F., PREBOIS, C. & ROCHEFORT, H. (1992). Inhibition of breast cancer growth by suramin. *J. Natl Cancer Inst.*, **84**, 38–42.